# TECHNOLOGY FOR HOME MONITORING

**DOI:** 10.1093/geroni/igz038.123

**Published:** 2019-11-08

**Authors:** Neil H Charness

**Affiliations:** Florida State University, Tallahassee, Florida, United States

## Abstract

Healthcare is increasingly moving away from expensive hospitals into the home. Although there are advantages to managing chronic health conditions in homes, there are also a number of disadvantages, beginning with aging adults’ unfamiliarity with the technologies being deployed. Too often, designs and instructional materials are not developed with aging users’ capabilities in mind. Health technology also confronts users with significant challenges including just-in-time learning when users are under significant stress. Technology support is often lacking. Also, care coordination for multiple chronic conditions is challenging. For instance, patients are often provided with multiple electronic health record portals. I discuss the reliability of and some of the practical challenges for a home health monitoring system used by older adults and those with heart failure over a 6-month interval. The system featured components such as a wrist-worn sensor package, daily tablet surveys, blood pressure cuff, weight scale, and bed sensors.

